# Biphenyl-4-yl 2,2,2-trichloro­ethyl sulfate

**DOI:** 10.1107/S1600536810012845

**Published:** 2010-04-14

**Authors:** Xueshu Li, Sean Parkin, Michael W. Duffel, Larry W. Robertson, Hans-Joachim Lehmler

**Affiliations:** a Department of Occupational and Environmental Health, University of Iowa, 100 Oakdale Campus, 124 IREH, Iowa City, IA 52242-5000, USA; b Department of Chemistry, University of Kentucky, Lexington, KY 40506-0055, USA; c College of Pharmacy, Division of Medicinal and Natural Products Chemistry, University of Iowa, Iowa City, IA 52242, USA

## Abstract

The mol­ecular structure of the title compound, C_14_H_11_Cl_3_O_4_S, displays a biphenyl dihedral angle of 4.9 (2)° between the benzene rings, which is significantly smaller than the calculated dihedral angle of 41.2° of biphenyl derivatives without *ortho* substituents. The C_Ar_—O bond length of 1.432 (4) Å is comparable with other sulfuric acid biphenyl-4-yl ester 2,2,2-trichloro­ether ester derivatives without electronegative substituents in the sulfated phenyl ring.

## Related literature

For similar structures of chlorinated sulfuric acid biphenyl-4-yl ester 2,2,2-trichloro-ethyl esters, see: Li *et al.* (2008 ▶, 2010 ▶). For a review of structures of sulfuric acid aryl mono esters, see: Brandao *et al.* (2005 ▶). For additional background information, see: Cravedi *et al.* (1999 ▶); Letcher *et al.* (2000 ▶); Liu *et al.* (2006 ▶, 2009 ▶); Ohnishi *et al.* (2000 ▶, 2001 ▶); Sacco & James (2005 ▶); Tampal *et al.* (2002 ▶); Robertson & Hansen (2001 ▶); Trotter (1961 ▶); Umeda *et al.* (2002 ▶, 2005 ▶). For further discussion of dihedral angles in chlorinated biphenyls, see: Shaikh *et al.* (2008 ▶).
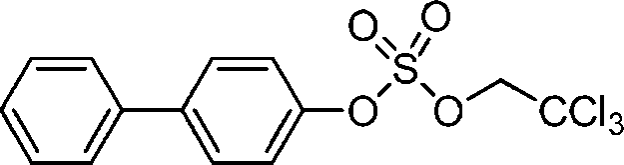


## Experimental

### 

#### Crystal data

C_14_H_11_Cl_3_O_4_S
*M* *_r_* = 381.64
Monoclinic, 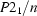

*a* = 7.5761 (2) Å
*b* = 5.8272 (2) Å
*c* = 35.2679 (11) Å
β = 90.181 (2)°
*V* = 1556.98 (8) Å^3^
*Z* = 4
Mo *K*α radiation
μ = 0.74 mm^−1^
*T* = 90 K
0.43 × 0.40 × 0.08 mm

#### Data collection

Nonius KappaCCD diffractometer
Absorption correction: multi-scan (*SADABS*; Bruker Nonius, 2006 ▶) *T*_min_ = 0.699, *T*_max_ = 0.944
15429 measured reflections
3041 independent reflections
1939 reflections with *I* > 2σ(*I*)
*R*_int_ = 0.079

#### Refinement

*R*[*F* ^2^ > 2σ(*F* ^2^)] = 0.054
*wR*(*F* ^2^) = 0.137
*S* = 1.09
3041 reflections
199 parameters
H-atom parameters constrained
Δρ_max_ = 0.62 e Å^−3^
Δρ_min_ = −0.46 e Å^−3^

### 

Data collection: *COLLECT* (Nonius, 1998 ▶); cell refinement: *SCALEPACK* (Otwinowski & Minor, 1997 ▶); data reduction: *DENZO-SMN* (Otwinowski & Minor, 1997 ▶); program(s) used to solve structure: *SHELXS97* (Sheldrick, 2008 ▶); program(s) used to refine structure: *SHELXL97* (Sheldrick, 2008 ▶); molecular graphics: *XP* in *SHELXTL* (Sheldrick, 2008 ▶); software used to prepare material for publication: *SHELXL97* and local procedures.

## Supplementary Material

Crystal structure: contains datablocks I, global. DOI: 10.1107/S1600536810012845/om2330sup1.cif

Structure factors: contains datablocks I. DOI: 10.1107/S1600536810012845/om2330Isup2.hkl

Additional supplementary materials:  crystallographic information; 3D view; checkCIF report

## Figures and Tables

**Fig. 1.**
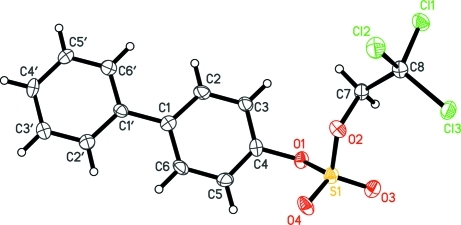
View of the title compound showing the atom-labeling scheme. Displacement ellipsoids are drawn at the 50% probability level.

## supplementary crystallographic information

### Comment

Exposure to biphenyl and structurally related chlorinated biphenyls has been associated with a range of adverse human health effects, including cancer and arteriosclerosis (Robertson & Hansen, 2001; Umeda *et al.*, 2005, 2002; Letcher *et al.*, 2000). Biphenyl and many lower chlorinated biphenyls are metabolized via hydroxylated biphenyl metabolites to sulfuric acid esters (Liu *et al.*, 2006, 2009; Ohnishi *et al.*, 2000, 2001; Sacco & James, 2005) and glucuronide conjugates (Cravedi *et al.*, 1999; Tampal *et al.*, 2002). While currently little is known about the toxicity of sulfate conjugates of chlorinated biphenyls, it is well established that sulfuric acid biphenyl-4-yl ester is involved in the formation of urinary calculi and, thus, plays a role in the induction of urinary bladder cancer (Ohnishi *et al.*, 2000, 2001). Unfortunately, crystal structures of (chlorinated) sulfuric acid biphenyl-4-yl esters have not been reported, partly because of their chemical instability (Li *et al.*, 2010). Here we report the crystal structure of a structurally related sulfuric acid biphenyl-4-yl ester 2,2,2-trichloro-ether ester.

In particular the C_Ar_—O bond length of sulfuric acid mono- and diesters may be predictive of the stability of the corresponding sulfuric acid conjugates (Brandao *et al.*, 2005; Li *et al.*, 2010). The C_Ar_—O (i.e. C4—O1) bond length of the title compound is 1.432 (4) Å, which is comparable to other, chlorinated sulfuric acid biphenyl-4-yl ester 2,2,2-trichloro-ether esters with no chlorine substituents in the sulfated benzene moiety (1.426 to 1.435 Å) (Li *et al.*, 2010, 2008). In contrast, the C_Ar_—O bond of sulfuric acid 2',3,5,5'-tetrachloro-biphenyl-4-yl ester 2,2,2-trichloro-ethyl ester, an analogous sulfuric acid diester with two chlorine substituents in the sulfated benzene moiety, is slightly shorter (1.405 (4) Å) due to the presence of the electronegative chlorine substituents (Li *et al.*, 2010). Therefore, the sulfuric acid biphenyl-4-yl ester corresponding to the title compound is expected to be relatively stable under physiological conditions, especially compared to aromatic sulfuric acid esters with electronegative substituents in the sulfated benzene ring.

The dihedral angle of biphenyl derivatives is associated with their affinity for cellular target molecules and, therefore, can correlate with their toxicity. The title compound adopts an almost planar conformation, with a solid state dihedral angle of the biphenyl moiety of 4.9 (2)°. Similarly, the parent compound, biphenyl, adopts a planar confirmation in the solid state with a dihedral angle of 0° (Trotter, 1961). These solid state dihedral angles are significantly smaller compared to the calculated dihedral angle of 41.2° of biphenyl derivatives without *ortho *substituents (Shaikh *et al.*, 2008). These deviations from the energetically most favorable conformation are most likely due to crystal packing effects, which allow the title compound to adopt an energetically less favorable conformation in the solid state by maximizing the lattice energy.

### Experimental

The title compound was synthesized from biphenyl-4-ol and 2,2,2-trichloroethyl sulfonyl chloride using 4-dimethylaminopyridine as catalyst (Li *et al.*, 2008). Crystals of the title compound suitable for crystal structure analysis were obtained by slow evaporation of a solution of the title compound in methanol.

### Refinement

H atoms were found in difference Fourier maps and subsequently placed in idealized positions with constrained C—H distances of 0.99 Å (CH_2_) and 0.95 Å (C_Ar_H) with *U*_iso_(H) values set to 1.2*U*_eq_ of the attached C atom.

### Crystal data

**Table:**

C_14_H_11_Cl_3_O_4_S	*F*(000) = 776
*M**_r_* = 381.64	*D*_x_ = 1.628 Mg m^−^^3^
Monoclinic, *P*2_1_/*n*	Mo *K*α radiation, λ = 0.71073 Å
Hall symbol: -P 2yn	Cell parameters from 19102 reflections
*a* = 7.5761 (2) Å	θ = 1.0–27.5°
*b* = 5.8272 (2) Å	µ = 0.74 mm^−^^1^
*c* = 35.2679 (11) Å	*T* = 90 K
β = 90.181 (2)°	Slab, colourless
*V* = 1556.98 (8) Å^3^	0.43 × 0.40 × 0.08 mm
*Z* = 4	

### Data collection

**Table:**

Nonius KappaCCD diffractometer	3041 independent reflections
Radiation source: fine-focus sealed tube	1939 reflections with *I* > 2σ(*I*)
graphite	*R*_int_ = 0.079
Detector resolution: 18 pixels mm^-1^	θ_max_ = 26.0°, θ_min_ = 2.3°
ω scans at fixed χ = 55°	*h* = −9→9
Absorption correction: multi-scan (*SADABS*; Bruker Nonius, 2006)	*k* = −7→7
*T*_min_ = 0.699, *T*_max_ = 0.944	*l* = −43→43
15429 measured reflections	

### Refinement

**Table:**

Refinement on *F*^2^	Primary atom site location: structure-invariant direct methods
Least-squares matrix: full	Secondary atom site location: difference Fourier map
*R*[*F*^2^ > 2σ(*F*^2^)] = 0.054	Hydrogen site location: inferred from neighbouring sites
*wR*(*F*^2^) = 0.137	H-atom parameters constrained
*S* = 1.09	*w* = 1/[σ^2^(*F*_o_^2^) + (0.0617*P*)^2^ + 1.446*P*] where *P* = (*F*_o_^2^ + 2*F*_c_^2^)/3
3041 reflections	(Δ/σ)_max_ = 0.001
199 parameters	Δρ_max_ = 0.62 e Å^−^^3^
0 restraints	Δρ_min_ = −0.46 e Å^−^^3^

### Special details

**Table:**

Geometry. All esds (except the esd in the dihedral angle between two l.s. planes) are estimated using the full covariance matrix. The cell esds are taken into account individually in the estimation of esds in distances, angles and torsion angles; correlations between esds in cell parameters are only used when they are defined by crystal symmetry. An approximate (isotropic) treatment of cell esds is used for estimating esds involving l.s. planes.
Refinement. Refinement of *F*^2^ against ALL reflections. The weighted *R*-factor wR and goodness of fit *S* are based on *F*^2^, conventional *R*-factors *R* are based on *F*, with *F* set to zero for negative *F*^2^. The threshold expression of *F*^2^ > 2σ(*F*^2^) is used only for calculating *R*-factors(gt) etc. and is not relevant to the choice of reflections for refinement. *R*-factors based on *F*^2^ are statistically about twice as large as those based on *F*, and *R*- factors based on ALL data will be even larger.

### Fractional atomic coordinates and isotropic or equivalent isotropic displacement parameters (Å^2^)

**Table:**

	*x*	*y*	*z*	*U*_iso_*/*U*_eq_	
S1	0.57446 (13)	0.92450 (18)	0.59262 (3)	0.0208 (3)	
O1	0.6710 (3)	0.7616 (5)	0.62196 (7)	0.0220 (7)	
O2	0.3875 (3)	0.8117 (5)	0.58864 (7)	0.0209 (6)	
O3	0.6713 (4)	0.8999 (5)	0.55880 (7)	0.0261 (7)	
O4	0.5421 (4)	1.1419 (5)	0.60895 (7)	0.0261 (7)	
Cl1	0.21565 (14)	0.28054 (18)	0.52992 (3)	0.0281 (3)	
Cl2	0.03163 (13)	0.6724 (2)	0.56132 (3)	0.0289 (3)	
Cl3	0.29160 (14)	0.73955 (18)	0.50317 (3)	0.0260 (3)	
C1	0.5167 (5)	0.7417 (7)	0.73623 (11)	0.0193 (9)	
C2	0.4726 (5)	0.5655 (7)	0.71089 (11)	0.0234 (10)	
H2	0.4087	0.4365	0.7199	0.028*	
C3	0.5198 (6)	0.5746 (7)	0.67315 (10)	0.0248 (10)	
H3	0.4888	0.4539	0.6563	0.030*	
C4	0.6121 (5)	0.7612 (7)	0.66051 (10)	0.0192 (9)	
C5	0.6597 (5)	0.9377 (8)	0.68381 (11)	0.0262 (10)	
H5	0.7243	1.0650	0.6743	0.031*	
C6	0.6116 (5)	0.9270 (7)	0.72177 (11)	0.0242 (10)	
H6	0.6441	1.0489	0.7383	0.029*	
C7	0.3776 (5)	0.5798 (7)	0.57323 (10)	0.0202 (9)	
H7A	0.3497	0.4696	0.5937	0.024*	
H7B	0.4928	0.5364	0.5621	0.024*	
C8	0.2351 (5)	0.5715 (7)	0.54294 (10)	0.0203 (9)	
C1'	0.4713 (5)	0.7278 (7)	0.77748 (10)	0.0188 (9)	
C2'	0.5201 (6)	0.9018 (7)	0.80282 (11)	0.0268 (10)	
H2'	0.5773	1.0352	0.7934	0.032*	
C3'	0.4867 (6)	0.8833 (8)	0.84125 (12)	0.0304 (11)	
H3'	0.5219	1.0033	0.8579	0.037*	
C4'	0.4027 (5)	0.6923 (8)	0.85568 (11)	0.0265 (10)	
H4'	0.3811	0.6788	0.8821	0.032*	
C5'	0.3503 (6)	0.5199 (8)	0.83080 (11)	0.0330 (11)	
H5'	0.2903	0.3887	0.8402	0.040*	
C6'	0.3851 (5)	0.5385 (8)	0.79256 (11)	0.0305 (11)	
H6'	0.3489	0.4184	0.7761	0.037*	

### Atomic displacement parameters (Å^2^)

**Table:**

	*U*^11^	*U*^22^	*U*^33^	*U*^12^	*U*^13^	*U*^23^
S1	0.0235 (6)	0.0200 (6)	0.0188 (5)	0.0022 (5)	−0.0014 (4)	0.0011 (4)
O1	0.0250 (16)	0.0222 (17)	0.0187 (14)	0.0051 (13)	−0.0025 (12)	0.0031 (12)
O2	0.0179 (15)	0.0206 (16)	0.0242 (15)	0.0040 (13)	−0.0021 (11)	−0.0042 (12)
O3	0.0283 (16)	0.0268 (18)	0.0232 (14)	0.0012 (14)	0.0034 (12)	0.0018 (13)
O4	0.0368 (17)	0.0170 (16)	0.0245 (15)	0.0058 (14)	−0.0058 (13)	−0.0071 (13)
Cl1	0.0364 (6)	0.0211 (6)	0.0267 (5)	−0.0025 (5)	−0.0040 (5)	−0.0021 (4)
Cl2	0.0223 (6)	0.0371 (7)	0.0274 (6)	0.0027 (5)	0.0009 (4)	−0.0017 (5)
Cl3	0.0353 (6)	0.0243 (6)	0.0186 (5)	−0.0011 (5)	0.0026 (4)	0.0022 (4)
C1	0.014 (2)	0.021 (2)	0.023 (2)	0.0037 (18)	−0.0018 (16)	0.0023 (18)
C2	0.032 (2)	0.011 (2)	0.027 (2)	−0.0016 (19)	−0.0028 (18)	0.0027 (18)
C3	0.038 (3)	0.018 (2)	0.019 (2)	−0.004 (2)	−0.0042 (19)	0.0019 (18)
C4	0.018 (2)	0.022 (2)	0.0171 (19)	0.0096 (18)	−0.0041 (16)	−0.0004 (18)
C5	0.024 (2)	0.029 (3)	0.026 (2)	−0.009 (2)	−0.0031 (18)	0.0021 (19)
C6	0.022 (2)	0.026 (3)	0.024 (2)	−0.0067 (19)	−0.0041 (17)	−0.0071 (19)
C7	0.028 (2)	0.015 (2)	0.0177 (19)	0.0027 (18)	−0.0002 (17)	−0.0003 (16)
C8	0.024 (2)	0.016 (2)	0.021 (2)	0.0034 (18)	0.0002 (17)	−0.0008 (17)
C1'	0.016 (2)	0.018 (2)	0.022 (2)	−0.0012 (18)	−0.0009 (16)	−0.0024 (17)
C2'	0.034 (3)	0.022 (3)	0.024 (2)	−0.003 (2)	0.0029 (19)	0.0009 (19)
C3'	0.037 (3)	0.030 (3)	0.024 (2)	−0.004 (2)	0.005 (2)	−0.006 (2)
C4'	0.026 (2)	0.034 (3)	0.019 (2)	0.005 (2)	0.0074 (18)	−0.0019 (19)
C5'	0.043 (3)	0.030 (3)	0.026 (2)	−0.013 (2)	0.012 (2)	−0.001 (2)
C6'	0.031 (3)	0.031 (3)	0.030 (2)	−0.009 (2)	0.006 (2)	−0.008 (2)

### Geometric parameters (Å, °)

**Table:**

S1—O3	1.410 (3)	C5—C6	1.390 (5)
S1—O4	1.414 (3)	C5—H5	0.9500
S1—O2	1.567 (3)	C6—H6	0.9500
S1—O1	1.582 (3)	C7—C8	1.518 (5)
O1—C4	1.432 (4)	C7—H7A	0.9900
O2—C7	1.459 (5)	C7—H7B	0.9900
Cl1—C8	1.762 (4)	C1'—C6'	1.389 (6)
Cl2—C8	1.774 (4)	C1'—C2'	1.400 (5)
Cl3—C8	1.765 (4)	C2'—C3'	1.384 (5)
C1—C6	1.395 (6)	C2'—H2'	0.9500
C1—C2	1.401 (5)	C3'—C4'	1.380 (6)
C1—C1'	1.498 (5)	C3'—H3'	0.9500
C2—C3	1.380 (5)	C4'—C5'	1.391 (6)
C2—H2	0.9500	C4'—H4'	0.9500
C3—C4	1.368 (6)	C5'—C6'	1.379 (5)
C3—H3	0.9500	C5'—H5'	0.9500
C4—C5	1.364 (5)	C6'—H6'	0.9500
			
O3—S1—O4	121.83 (18)	C8—C7—H7A	109.9
O3—S1—O2	110.75 (16)	O2—C7—H7B	109.9
O4—S1—O2	104.74 (16)	C8—C7—H7B	109.9
O3—S1—O1	104.57 (16)	H7A—C7—H7B	108.3
O4—S1—O1	110.58 (15)	C7—C8—Cl1	105.8 (3)
O2—S1—O1	102.89 (15)	C7—C8—Cl3	111.6 (3)
C4—O1—S1	118.5 (2)	Cl1—C8—Cl3	110.3 (2)
C7—O2—S1	117.8 (2)	C7—C8—Cl2	110.5 (3)
C6—C1—C2	117.1 (4)	Cl1—C8—Cl2	110.0 (2)
C6—C1—C1'	121.1 (4)	Cl3—C8—Cl2	108.6 (2)
C2—C1—C1'	121.7 (4)	C6'—C1'—C2'	117.0 (4)
C3—C2—C1	121.7 (4)	C6'—C1'—C1	121.6 (4)
C3—C2—H2	119.2	C2'—C1'—C1	121.3 (4)
C1—C2—H2	119.2	C3'—C2'—C1'	121.3 (4)
C4—C3—C2	118.6 (4)	C3'—C2'—H2'	119.3
C4—C3—H3	120.7	C1'—C2'—H2'	119.3
C2—C3—H3	120.7	C4'—C3'—C2'	120.7 (4)
C5—C4—C3	122.5 (4)	C4'—C3'—H3'	119.7
C5—C4—O1	119.2 (4)	C2'—C3'—H3'	119.7
C3—C4—O1	118.2 (3)	C3'—C4'—C5'	118.7 (4)
C4—C5—C6	118.5 (4)	C3'—C4'—H4'	120.6
C4—C5—H5	120.8	C5'—C4'—H4'	120.6
C6—C5—H5	120.8	C6'—C5'—C4'	120.4 (4)
C5—C6—C1	121.6 (4)	C6'—C5'—H5'	119.8
C5—C6—H6	119.2	C4'—C5'—H5'	119.8
C1—C6—H6	119.2	C5'—C6'—C1'	121.9 (4)
O2—C7—C8	109.1 (3)	C5'—C6'—H6'	119.1
O2—C7—H7A	109.9	C1'—C6'—H6'	119.1
			
O3—S1—O1—C4	175.2 (3)	C1'—C1—C6—C5	177.7 (4)
O4—S1—O1—C4	42.4 (3)	S1—O2—C7—C8	−132.5 (3)
O2—S1—O1—C4	−69.0 (3)	O2—C7—C8—Cl1	−173.5 (2)
O3—S1—O2—C7	48.4 (3)	O2—C7—C8—Cl3	66.5 (3)
O4—S1—O2—C7	−178.5 (2)	O2—C7—C8—Cl2	−54.5 (4)
O1—S1—O2—C7	−62.8 (3)	C6—C1—C1'—C6'	−176.6 (4)
C6—C1—C2—C3	−0.4 (6)	C2—C1—C1'—C6'	0.7 (6)
C1'—C1—C2—C3	−177.7 (4)	C6—C1—C1'—C2'	1.0 (6)
C1—C2—C3—C4	0.1 (6)	C2—C1—C1'—C2'	178.2 (4)
C2—C3—C4—C5	0.3 (6)	C6'—C1'—C2'—C3'	1.2 (6)
C2—C3—C4—O1	175.7 (3)	C1—C1'—C2'—C3'	−176.4 (4)
S1—O1—C4—C5	−77.2 (4)	C1'—C2'—C3'—C4'	−0.5 (7)
S1—O1—C4—C3	107.2 (4)	C2'—C3'—C4'—C5'	−0.8 (7)
C3—C4—C5—C6	−0.3 (6)	C3'—C4'—C5'—C6'	1.2 (7)
O1—C4—C5—C6	−175.7 (3)	C4'—C5'—C6'—C1'	−0.5 (7)
C4—C5—C6—C1	−0.1 (6)	C2'—C1'—C6'—C5'	−0.8 (6)
C2—C1—C6—C5	0.4 (6)	C1—C1'—C6'—C5'	176.9 (4)
